# Capture-recapture method for assessing publication bias

**Published:** 2010

**Authors:** Jalal Poorolajal, Ali Akbar Haghdoost, Mahmood Mahmoodi, Reza Majdzadeh, Siavosh Nasseri-Moghaddam, Akbar Fotouhi

**Affiliations:** a Department of Epidemiology and Biostatistics, School of Public Health, Tehran University of Medical Sciences, Tehran, Iran; b Community Medicine Department and Physiology Research Center, Kerman University of Medical Sciences, Kerman, Iran; c Digestive Disease Research Center, Tehran University of Medical Sciences, Tehran, Iran

**Keywords:** Meta-Analysis, Publication Bias, Review Literature, Capture-Recapture

## Abstract

**BACKGROUND:**

Publication bias is an important factor that may result in selection bias and lead to overestimation of the intervention effect. In this study, the focus was on using capture-recapture method as a statistical procedure which may possibly be a practical means for measuring the amount of publication bias.

**METHODS:**

A systematic review was conducted to estimate the duration of protection provided by hepatitis B vaccine by measuring the anamnestic immune response to booster doses of vaccine and retrieved studies from three separate sources of electronic databases, reference lists of the studies, and conference databases as well as contact with experts and manufacturers. Capture-recapture and some conventional methods such as funnel plot, Begg test, Egger test, and trim and fill method were employed for assessing publication bias.

**RESULTS:**

Based on capture-recapture method, completeness of the overall search results was 87.2% [95% CI: 84.6% to 89.0%] and log-linear model suggested 5 [95% CI: 4.2 to 6.2] missing studies. The funnel plot was asymmetric while Begg and Egger tests results were statistically insignificant and trill and fill approach made no change in pooled effect.

**CONCLUSIONS:**

Capture-recapture method may be a useful practical approach for estimating the number of missing studies which are not usually detected by search strategy. As a result, use of capture-recapture method as an alternative approach could be suggested for estimating the extent of publication bias based on overlapping information rather than mirror image of extreme values on funnel plot.

Capture-recapture method, called the Petersen method, has a very long history and is widely used in ecology to estimate the unknown size of wild animals’ population.^1^ Another important application for this method is in epidemiology for estimating prevalence of a particular disease and estimating the completeness of ascertainment of disease registers.^2^,^3^ However, capture-recapture method can principally be applied to any situation where there are two or even more incomplete lists. This method was recently used as a potentially useful method for estimating publication bias^4^ in systematic reviews where different sources are used to include as many references as possible but neither of sources of retrieving studies is complete.

This study focuses on using capturerecapture method as a statistical procedure which may possibly, but not necessarily, be a practical means for measuring the amount of publication bias by estimating the number of missing studies not identified by search strategy, but potentially eligible to be included in the systematic review in comparison with other conventional methods exploring publication bias.

## Methods

The simplest capture-recapture model is socalled 2-sample model. In the first sample, a group of individuals are captured for marking with a unique identifier, and then are released back to the population. In the second sample, there are some of the individuals caught and marked during the initial sampling and some new individuals caught in just the second sampling. It is possible to estimate the number individuals not caught in either samples, thus providing an estimate of the total population size.^1^

**Figure 1:**
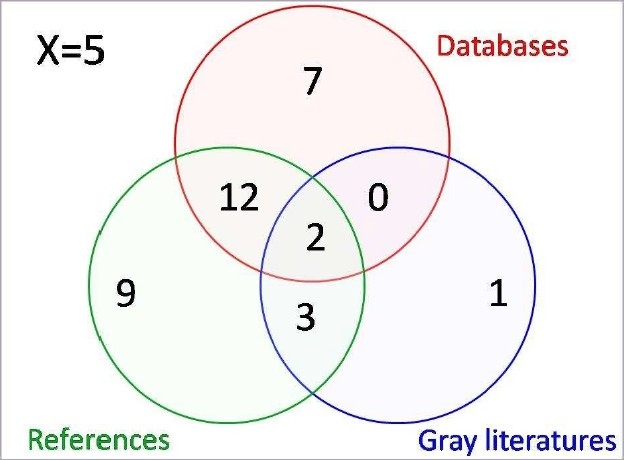
Distribution of the studies by sources of capture

In this study, the capability of capture-recapture method for assessing publication bias in a systematic review was explored. This systematic review was conducted to measure the anamnestic immune response to booster doses and to estimate the duration of protection provided by hepatitis B vaccine.^5^ In the review, both randomized and non-randomized studies were included, addressing anamnestic immune response (AIR) to booster of HB vaccine 5 years or more post primary vaccination in healthy participants vaccinated in a 3-dose or 4-dose schedule without receiving additional dose or immunoglobulin. In the review, three different sources were searched, including electronic databases, reference lists of studies, and unpublished data or so-called gray literatures including conference databases as well as personal contact with experts and manufacturers (Figure 1). The Cochrane Central Register of Controlled Trials (The Cochrane Library 2008, Issue 3), MEDLINE (Jan 1950 to Dec 2008), EMBASE (Jan 1980 to Dec 2008) and ISI (Jan 1945 to Dec 2008) were searched. The following conference databases up to December 2008 were also searched for unpublished data:

Annual Meeting of the Infectious Diseases Society of America (IDSA); available at: http://www.idsociety.org;

European Congress of Clinical Microbiology and Infectious Diseases (ECCMID); available at: http://www.escmid.org;

Interscience Conference on Antimicrobial Agents and Chemotherapy (ICAAC); available at: http://www.icaac.org.

In addition, the authors of included studies as well as vaccine manufacturers for additional unpublished trials were contacted.

**Figure 2:**
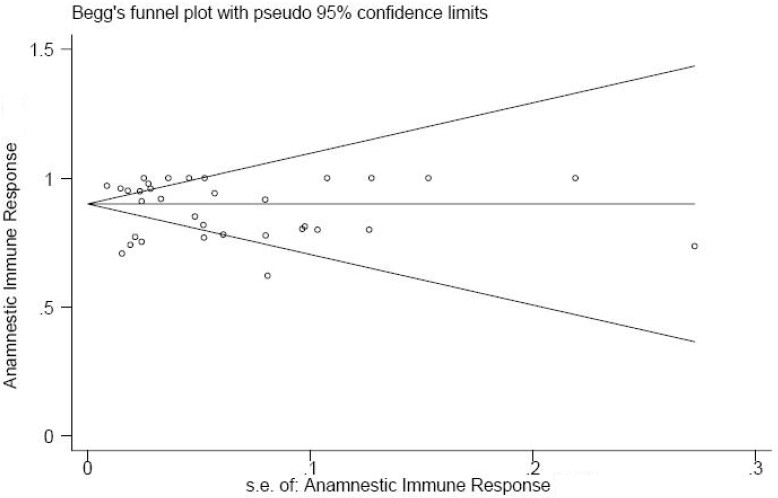
Forest plot of Anamnestic Immune Response (AIR) to booster dose in non-protected vaccinees

**Figure 3:**
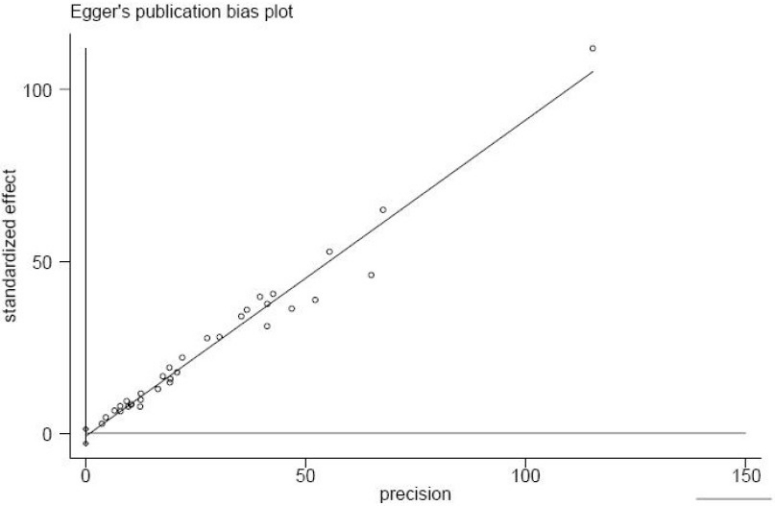
Funnel plot for of standard error of estimation against anamnestic immune response rate

Statistical heterogeneity was explored using the chi-squared (χ^2^ or Chi^2^) test at the 10% significance level (p < 0.10). Inconsistency across studies results was quantified using I^2^ statistic.^6^ Also, the between-study variance was estimated using tau-squared (τ^2^ or Tau^2^) statistic^7^ (Figure 2). The funnel plot was used to assess publication bias (Figure 3).

By December 2008, 4699 references were retrieved, including 2208 references through searching electronic databases, 2467 references through checking reference lists, and 24 references through personal contact with studies’ authors or searching conference databases. Having checked the eligibility of references, 34 studies involving 4479 individuals were eventually included in the review (Figure 2). According to results of the review, the vaccine’s protection is mostly dependent on immune memory rather than anti-HBs, hence, booster doses should be recommended based on immune memory, rather than persistence of antibody. Besides, a full course of HB vaccination can induce a long-term and strong serologic immunity against HBV infection. However, the decreasing trend of seroprotection during the first and second decades after immunization indicates that the long-term immunity induced by the HB vaccine may diminish over time. This issue may raise the possible need for booster dose, although universal revaccination does not seem necessary during the first and second decade post primary vaccination in healthy individuals with normal immune status, who have fully responded to a complete course of vaccine. The more details of seroprotection of HB vaccine and need for booster dose as well as long-term protection provided by HB vaccine are reported elsewhere.^5^,^8^

As mentioned before, three different data sources were used for retrieving eligible studies in the review but none of the data sources was complete. In such a situation, there might be studies included in none of sources so-called missing studies. However, it is possible to estimate the missing studies using the 3-sample capture-recapture method. When there are three sources, the capture-recapture method becomes more complicated, including the following 8 possible models:

1. number of studies identified by databases only (A)
2. number of studies identified by reference lists only (B)
3. number of studies identified by personal contact (so called gray literatures) (C)
4. number of studies identified by sources A and B but not by source C (AB)
5. number of studies identified by sources A and C but not by source B (AC)
6. number of studies identified by sources B and C but not by source A (BC)
7. number of studies identified by all three sources (ABC)
8. number of missing studies identified by none of the three sources (X)

There are many elaborate statistical models available for the analysis of 3-source capture-recapture results. Log-linear is a simple model which easily accommodates the three sources and is able to explore dependence between sources and adjust for it by including interaction terms in the model.^9^ In addition, based on the above available information, log-linear model can be applied to estimate the number of studies not identified by all three sources (X) and hence the total probably eligible studies (N).

There are two main information criteria proposed for model selection, including Akaike’s Information Criterion (AIC) and Bayesian Information Criterion (BIC).^10^ The AIC is calculated as:

AIC = G^2^ – [2 × (df)]

Where G^2^ is the likelihood ratio statistic associated with the fit of any model to the data, and df is the degree of freedom of the model. The model giving the smallest value of AIC is the one selected.^10^,^11^

The second criterion, BIC, is preferred to AIC in some applications and is as follows:

BIC = G^2^ – [ln (N_obs_/2π)] × (df)

With G^2^ and df as above, and ln N_obs_ is natural logarithm of the observed sample size.

The results of using capture-recapture method for assessing publication bias and estimation of missing studies were compared with other conventional methods including: the Begg adjusted rank correlation test, the Egger regression asymmetry test and the Duval and Tweedie nonparametric ‘trim and fill’ method used for exploring publication bias, and advantages and disadvantages of each method were discussed. The statistical package Stata 9 and Revman 5, comprehensive package for systematic review, was used for data analysis.

## Results

Out of 34 studies obtained from three different sources, 50% of studies were identified at least by 2 sources and 6% by all three sources (Figure 1). The log-linear model revealed no statistically significant interaction or positive dependence between three sources (Table 1). The first model (no interaction model) was the best fit model that had the smallest value of AIC and BIC. According to these findings, 5 [95% CI: 4.2 to 6.2] studies were estimated to be probably eligible but not identified by the search strategy. Hence, the completeness of the overall search results was 87.2% [95% CI: 84.6% to 89.0%]. Based on these results, checking reference lists was more complete and hence more sensitive for finding references than the other two sources (Table 2).

**Table 1:** Log-linear models fitted to three sources of search strategy and estimated number of eligible studies

						95% CI			
Model	Df*	G^2^**	P***	N_est_†	X††	Lower	Upper	AIC†††	BIC§
A B C	3	3.10	0.3767	39	5	4.2	6.2	-2.90	-8.83
A B C AB	2	3.09	0.2131	40	6	3.4	7.8	-0.91	-4.87
A B C AC	2	1.84	0.3992	38	4	3.2	5.3	-2.16	-6.12
A B C BC	2	1.98	0.3715	41	7	5.4	7.6	-2.02	-5.98
A B C AB AC	1	1.73	0.1890	37	3	0.6	5.4	-0.27	-2.25
A B C AB BC	1	0.48	0.4896	∞	∞	-	-	-1.52	-3.50
A B C AC BC	1	0.91	0.3406	39	5	4.1	6.4	-1.09	-3.07
A B C AB AC BC	0	0.00	1.0	∞	∞	-	-	0.00	0.00

* df: degree of freedom

** G^2^: likelihood ratio statistic

*** P: p value

† N_est_: estimated total number

†† X: unknown data

††† AIC: Akaike’s Information Criterion

§ BIC: Bayesian Information Criterion

**Table 2:** Comprehensiveness of the three sources

				95% CI %	
Sources	n (observed)	n (estimated)	Completeness (%)	Lower	Upper
Databases	21	39	53.8	52.2	54.9
Reference lists	26	39	66.7	64.7	68.1
Personal contact	6	39	15.4	14.9	15.7
All three sources	34	39	87.2	84.6	89.0

**Figure 4:**
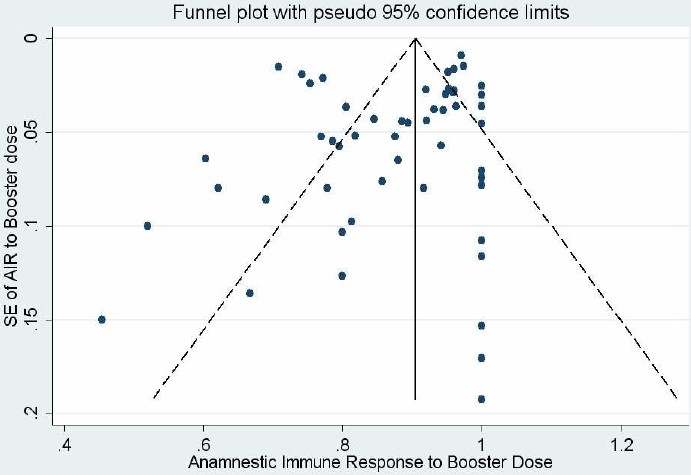
Begg’s adjusted rank correlation funnel plot of standard error of estimation against anamnestic immune response rate

**Figure 5:**
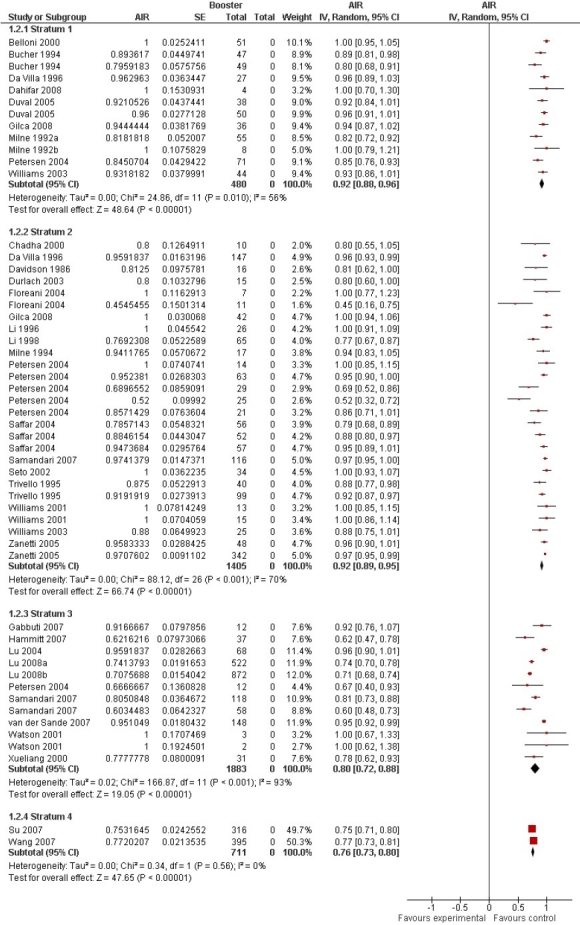
Egger’s regression asymmetry plot of standard error of estimation against anamnestic immune response rate

In this study, the Begg adjusted rank correlation test for publication bias was applied (Figure 4). The spread of results was the same at all values of the sample around the middle line but the studies were distributed mostly at the narrower side of the funnel and the plot was reasonably asymmetrical. However, the result of Begg test was not statistically significant (p = 0.374). In addition, publication bias was explored using the Egger regression asymmetry test (Figure 5). The regression line passed through the origin and the test results was not statistically significant (p = 0.379).

Also publication bias was investigated using the Duval and Tweedie nonparametric “trim and fill” method which allows estimation of adjusted meta-analysis. However the “trim and fill” procedure suggested no missing study to the funnel plot in the present review and made no change in meta-analysis results and hence indicated no evidence of publication bias.

## Discussion

Capture-recapture method represents an attractive approach to investigate the completeness of search strategy results and to quantify the amount of publication bias by estimating the number of missing studies which might be eligible but were virtually not included in a systematic review.

Although capture-recapture approach is a potential useful method for estimating the number of unknown studies which are not usually identified by search strategy, however, this method, like any other statistical procedures, has its own limitations. A critical limitation of this method is that sufficiently high overlapping information is required to produce reliable estimate of missing studies. Otherwise, the likelihood functions may become flat and the resulting estimates based on log-linear models may possibly become unstable.^9^ Another limitation of capture-recapture method using log-linear model for investigating publication bias is that relative large number of studies is required to hold the assumption of the normal distribution within log-linear models, whereas this assumption is not usually met because of limited number of studies in most systematic reviews. The third limitation is that capture-recapture method is not able to correct and adjust the pooled estimate for publication bias as trim and fill is. Moreover, validity of capture-recapture results depends on some assumptions. If the assumptions are not considered, the estimates may not be reliable. A critical assumption of capture-recapture methods is the independence of the sources so that either positively or negatively dependent sources may cause either underestimation or overestimation of the pooled estimates respectively.^1^ Of course, log-linear model is able to handle dependence among sources and adjust for it by including interaction terms in the model.^9^

It is important to keep in mind, however, that capture-recapture is a useful method for estimating missing studies detected by none of data sources, but it is rather different from the concept of publication bias. In other words, methods of exploring publication bias like the trim and fill method is built on the strong assumption that there should be a symmetric funnel plot. Indeed, the trim and fill method provides an estimate of the number of missing studies as well as an adjusted intervention effect for the publication bias based on the filled studies.^7^ Hence it is possible that the trim and fill method find no publication bias in the presence of a relatively symmetric funnel plot while capture-recapture method may suggest considerable number of missing studies.

On the other hand, the funnel plot is a simple graphical approach which is frequently used for assessing publication bias. However, the visual interpretation of funnel plots is too subjective and researchers have limitation to identify the amount of publication bias quantitatively.^7^ In addition, funnel plot asymmetry may raise the possibility of publication bias but it does not prove it.^12^

The Begg adjusted rank correlation test and the Egger regression asymmetry graph are statistical techniques for exploring the publication bias. Nonetheless, neither Begg test nor Egger test revealed a significant publication bias because both techniques have low power for detecting publication bias, although the regression method appeared more sensitive than the rank correlation method and tend to suggest the presence of publication bias more frequently than the Begg approach.^13^

The trim and fill method is a useful approach for estimation of an adjusted pooled effect and hence sensitivity analysis of the presence of publication bias. However, this procedure suggested no missing study to the funnel plot in the present review and made no change in meta-analysis results and hence indicated no evidence of publication bias. The reason is that the performance of this method for detecting publication bias is poor especially when heterogeneity exists among the studies.^14^

As mentioned in the introduction, capture-recapture method was first used by Bennett et al as a potentially useful method for estimating publication bias.^4^ In their study, the number of missing studies estimated by capture-recapture method was much less than that estimated by the trim and fill approach, which was contrary to the present findings. In the review, the capture-recapture approach suggested 5 missing studies whereas trim and fill approach estimated no missing studies.

## Conclusions

Capture-recapture method is a useful practical approach for estimating the number of missing studies which are not usually identified by search strategy, although assumptions of this method may limit its general application in systematic reviews. In addition, capture-recapture method may be considered as an alternative approach for estimating the extent of publication bias based on overlapping information rather than mirror image of extreme values on funnel plot.
